# Routine Stent Placement in Endoscopic Management of Appendicitis: Necessity or Optional?

**DOI:** 10.1055/a-2882-1421

**Published:** 2026-07-13

**Authors:** Nini Cao, Hao-xin Chen, Ting-ting Liu, Jing Zhou, De-feng Li, Yang Song, Jun Yao

**Affiliations:** 1 Department of Gastroenterology, Shenzhen People’s Hospital, The Second Clinical Medical College 47885 Jinan UniversityShenzhenGuangdongChina

**Keywords:** endoscopic retrograde appendicitis therapy (ERAT), stent placement, acute uncomplicated appendicitis, acute suppurative appendicitis, periappendiceal abscess, chronic appendicitis

## Abstract

The primary goal of stent placement during endoscopic retrograde appendicitis therapy (ERAT) is to ensure effective drainage and alleviate luminal obstruction. Placement of a stent within the appendiceal lumen as a therapeutic intervention has been demonstrated to significantly reduce the risk of disease recurrence. Despite this, the routine use of drainage stents remains a subject of debate, and no standardized guidelines currently exist to direct their application. Consequently, the decision to deploy a drainage stent often relies on the operator’s clinical judgment. Within the context of ERAT for appendicitis, treatment can be stratified into three categories, each associated with distinct strategies regarding routine stent placement. This review synthesizes recent literature and offers a practical reference for clinicians considering stent deployment across these different scenarios.

## 1 Overview of Appendicitis Diagnosis and Treatment

Appendicitis is an acute abdominal disorder arising from inflammatory processes within the appendix and its surrounding tissues. Current management strategies include conservative antibiotic therapy, appendectomy, and endoscopic retrograde appendicitis therapy (ERAT). In recent years, the long-standing status of appendectomy as the “gold standard” for acute appendicitis has been increasingly questioned, largely due to the inherent risks of postoperative complications and the substantial rate of negative (normal) appendectomies. 1 In cases of uncomplicated acute appendicitis, some patients may choose conservative antibiotic treatment to avoid surgical trauma and preserve the physiologic function of the appendix. However, this approach is often associated with prolonged hospitalization and a considerable risk of recurrence, with approximately one-third of patients initially treated with antibiotics ultimately requiring appendectomy. 2 Notably, antibiotic therapy alone shows limited efficacy in patients with an embedded appendiceal fecalith, leading to higher appendectomy rates and greater susceptibility to complications, including surgical site and intraabdominal infections. 3

Advances in endoscopic technology have introduced ERAT as a novel therapeutic alternative. This technique enables direct access to the appendiceal orifice via colonoscopy, allowing for real-time visualization of inflammatory changes in the surrounding mucosa, including congestion, edema, or purulent exudate. Retrograde intubation further permits detailed imaging of the appendiceal lumen under fluoroscopy, facilitating the assessment of luminal filling, detection of radiolucent fecaliths, dilation, or stenosis, as well as key anatomic features such as wall smoothness, alignment distortion, and peristaltic function. By enabling precise diagnosis, ERAT reduces the likelihood of misdiagnosis, missed diagnosis, and unnecessary appendectomies.

Once appendicitis is confirmed, ERAT allows immediate endoscopic intervention, including luminal decompression, irrigation, fecalith extraction, and stent placement for drainage, thereby rapidly lowering intraluminal pressure and alleviating clinical symptoms. Modifications such as ultrasound (US)-guided ERAT enable real-time, radiation-free monitoring of intubation and flushing, which is particularly advantageous for vulnerable populations, including pregnant women and children. Additionally, the integration of direct choledochoscopic visualization systems provides endoscopic treatment under direct vision, further enhancing diagnostic accuracy and procedural safety.

Clinical studies indicate that ERAT reduces operative time by an average of 13 minutes, offers faster postoperative pain relief, and is associated with a lower incidence of adverse events compared with laparoscopic appendectomy, while hospital stay and patient satisfaction remain comparable. 4 Emerging evidence underscores that the appendix is not a vestigial organ but an immune-associated structure contributing to intestinal microbiota homeostasis and biofilm maintenance, with potential implications in the pathogenesis of ulcerative colitis. 5 Luminal obstruction by fecal debris, hyperplastic lymphoid tissue, parasites, or neoplasms is the primary mechanism underlying acute appendicitis, whereas chronic appendicitis often results from partial or persistent luminal obstruction. ERAT addresses these pathophysiologic processes by relieving appendiceal obstruction, draining purulent material, and removing fecaliths. In Ding et al.’s feasibility study on ERAT for acute appendicitis treatment, the recurrence rate of appendicitis in the ERAT group was 2.86% during the six-month postoperative follow-up period. Its therapeutic efficacy in acute uncomplicated appendicitis is well established 6 (randomized controlled trial, RCT, evidence grade 1B), and evidence also supports its feasibility and effectiveness in the management of periappendiceal abscesses and chronic appendicitis. 7 8

For chronic appendicitis, long-term luminal obstruction by fecaliths, adhesions, or benign stenosis leads to impaired drainage and recurrent inflammation. ERAT achieves direct visualization of the appendiceal lumen to identify lesions, then removes obstructions via basket lithotomy or balloon dilation, or places stents to dilate stenotic segments, restoring luminal patency and eliminating the root cause of repeated attacks.

For acute appendicitis, ERAT first reduces intraluminal pressure rapidly by irrigating the lumen with normal saline or antibiotic solutions to drain pus and inflammatory exudates, thus preventing infection spread. It then clears the obstructive factors causing acute inflammation and implants plastic stents for continuous drainage in severe cases, controlling acute inflammatory responses effectively.

## 2 Stent Placement for Different Types of Appendicitis

### 2.1 Acute Uncomplicated Appendicitis

The diagnosis of acute appendicitis typically relies on a combination of clinical history, presenting symptoms, physical examination, laboratory findings (such as elevated white blood cell count), and imaging studies. Approximately 70% of cases exhibit classic features, including migratory pain to the right lower quadrant, tenderness at McBurney’s point, and rebound pain. US and computed tomography (CT) remain the most commonly employed imaging modalities. However, in obese or pregnant patients, visualizing the appendix via US can be challenging. Notably, one study has reported that chronic inflammation is identified in 9% of patients whose diagnosis relies solely on CT imaging. 9

The endoscopic management of acute appendicitis was first explored by Said et al. in 1995, 10 who accessed an inflamed appendix and drained appendiceal pus using an endoscopic retrograde cholangiopancreatography (ERCP) catheter. Following endoscopic aspiration, patients experience near-immediate relief of clinical symptoms, with signs of inflammation resolving within a few days. This case underscores the critical role of reducing intra-appendiceal pressure in controlling acute inflammation, forming the basis of the ERAT protocol systematically described later by Liu et al.

In Liu’s follow-up cohort, seven patients have experienced recurrence, five of whom (71.4%) have undergone simple irrigation without stent placement, with a mean recurrence interval of 30 days. 11 The researchers have concluded that cannula suction and irrigation alone may not achieve complete drainage, whereas stent placement ensures continuous and sufficient drainage, thereby lowering recurrence risk. In a prospective comparison of ERAT versus appendectomy, Liu et al. have further observed that patients who initially experienced recurrence without stenting had no subsequent recurrence after stent placement. These findings support the notion that adequate luminal drainage, reinforced by stent insertion, can reduce recurrence rates. 12

Similarly, a retrospective analysis by Yang et al. 13 has corroborated these observations. In a case series reported by Tao et al., 14 all patients treated with ERAT did not receive postoperative stents; one patient ultimately underwent appendectomy on the second postoperative day due to persistent abdominal pain, with pathology confirming acute suppurative appendicitis. This instance highlights the importance of intraoperative drainage strategy in ERAT: leaving a stent in place may improve postoperative symptom control and reduce the likelihood of conversion to surgery due to unresolved pain.

While the majority of ERAT-related studies advocate routine stent placement, 15 some researchers suggest selective stenting only in cases with high pus volume or marked luminal narrowing to ensure adequate drainage. 4 12 13

### 2.2 Acute Suppurative Appendicitis or Periappendiceal Abscess

Acute suppurative appendicitis and periappendiceal abscess represent forms of complicated acute appendicitis. Acute suppurative appendicitis, also referred to as appendiceal cellulitis, is characterized by diffuse inflammation in the lower right quadrant of the appendix. When appendicitis progresses to septic, gangrenous, or perforated stages, the periappendiceal greater omentum may encapsulate the inflammatory exudate, forming a localized inflamed mass containing pus, known as a periappendiceal abscess. Current management typically involves early surgical intervention to prevent severe complications or conservative approaches combining antibiotics with percutaneous drainage guided by US or CT imaging. However, appendectomy carries inherent postoperative risks, including infection, intra-abdominal adhesions, and residual abscess formation.

Obstruction of the appendiceal lumen by fecaliths is a key pathophysiologic factor, leading to elevated intraluminal pressure, bacterial overgrowth, and exacerbation of suppurative inflammation, ultimately promoting abscess formation and delaying symptom resolution. 16 ERAT addresses both obstruction and infection: it drains the appendix while removing luminal blockages via mesh basket extraction, alleviating symptoms and potentially lowering recurrence rates. When combined with a short course of antibiotics and delayed appendectomy, ERAT may also shorten hospitalization and reduce readmission rates.

In a study by Ding et al., 17 13 patients with periappendiceal abscesses underwent hybrid ERAT treatment and were discharged without postoperative complications; no adverse events were observed during 6 months of follow-up, demonstrating the therapeutic advantages of ERAT in managing periappendiceal abscesses. Similarly, Liu et al. have reported six cases of periappendiceal abscess treated with appendiceal stenting and drainage, resulting in immediate pain relief and an average hospital stay of 5 days. 11 No complications or recurrence are observed during follow-up, highlighting the potential of stenting to shorten hospitalization while avoiding surgical morbidity.

Further evidence indicates that stent placement significantly reduces recurrence. Among 94 patients with acute suppurative appendicitis, 12 patients (12.8%) experienced recurrence within 1 year. In the stent group, only 2 patients (4.7%) recurred, compared with 10 patients (19.2%) in the stentless group, with stent retention time inversely correlated with recurrence. 6 Stent insertion serves to dilate inflamed, narrowed lumens; maintain patency; and prevent short-term postoperative fecalith obstruction. Consequently, indwelling appendiceal stents are now widely recognized as essential in the management of acute suppurative appendicitis and periappendiceal abscesses, particularly in reducing recurrence and improving clinical outcomes.

### 2.3 Chronic Appendicitis

Clinically, chronic appendicitis is defined by recurrent right lower abdominal pain persisting over months to years, which may intermittently worsen, but typically without peritoneal signs, and after exclusion of other causes of right lower quadrant pain. Histopathologically, chronic appendicitis is characterized by (1) a fourfold increase in lymphoid follicles; (2) activation of interfollicular reticular cells in the mucosa; (3) plasma cells constituting at least 20% of exudative cells; and (4) plasma cell infiltration extending to the muscularis propria and serosal layers. Mattei et al. 18 have proposed four clinical criteria for diagnosing chronic appendicitis: (1) right lower abdominal pain persisting for more than 3 weeks; (2) exclusion of alternative diagnoses explaining the symptoms; (3) histopathologic evidence of chronic inflammation or fibrosis in the appendix; and (4) complete symptom resolution after appendectomy.

Differentiating acute from chronic appendicitis is challenging, even when considering primary or secondary CT findings, which may include appendiceal dilation, periappendiceal fat stranding, fibrosis, wall thickening with peripheral edema, appendicoliths, abscess formation, and regional lymphadenopathy. 9 19 20 The atypical clinical presentation and nonspecific imaging features contribute to the difficulty of establishing an initial diagnosis.

ERAT has emerged as a safe, effective, and minimally invasive approach for both diagnosis and management of chronic appendicitis. 8 24 In most patients, inflammatory markers are within normal limits, and the appendiceal orifice appears macroscopically normal. In a retrospective study by Li et al., 8 60 patients were included, among whom 57 (95%) exhibited a normal appendiceal opening; 52 patients (86.7%) had congested and edematous appendiceal walls; 4 patients (6.7%) had appendiceal stenosis; and 45 patients (75%) had fecaliths or residual fecal matter within the lumen. To maintain continuous luminal patency and drainage, stents are placed in 20 patients (33.3%) and removed after 3 months, achieving a final clinical success rate of 90% (54/60).

Ma et al. 21 have recommended stent placement in the presence of infection, appendiceal orifice swelling, suspected residual stones, difficult-to-manage appendiceal calculi, luminal stenosis, or distortion. Clinical success is achieved in 91.7% of patients, with no short- or long-term adverse events reported in those who do not receive stents. This finding suggests that in the absence of these risk factors, luminal cleaning without stenting is both safe and effective. Logistic regression analysis further identifies appendiceal stenosis and luminal distortion as significant predictors of long-term adverse outcomes.

Several cases demonstrate that even patients with large or multiple fecaliths may not require stenting. For instance, a 28-year-old patient with multiple appendiceal stones has undergone ERAT without stent placement, experienced symptomatic improvement, and had no recurrence. 22 Similarly, a 13-year-old patient treated with ERAT without stenting has achieved complete symptom relief and remained recurrence-free. 23 Collectively, these findings indicate that stent placement to maintain luminal dilation and drainage is particularly beneficial in patients with severe inflammation or stenosis, reducing the likelihood of adverse events. 24 25

## 3 Outlook

As discussed, stent placement is now routinely performed in patients with acute suppurative appendicitis, periappendiceal abscess, and chronic appendicitis associated with severe inflammation or luminal stenosis. In contrast, its use in acute uncomplicated appendicitis remains a subject of debate. Recent advances in ERAT have not only enabled effective management of appendiceal inflammation but also preserved the physiologic integrity of the appendix and maintained its tubular continuity.

Nevertheless, prolonged stent retention carries potential risks, including foreign body reactions, bleeding, perforation, and secondary infections. Consequently, postplacement management requires careful consideration, with future strategies focusing on the timing of stent placement, optimal drainage, and duration of retention. In cases of acute uncomplicated appendicitis, most clinicians recommend stent removal within 1–2 weeks; if spontaneous dislodgement does not occur, removal via follow-up colonoscopy is advised. For patients with acute suppurative appendicitis, periappendiceal abscess, or chronic appendicitis, stents typically dislodge spontaneously or are removed endoscopically within three months.

Despite these clinical advancements, the broader application of ERAT is constrained by limitations in the current evidence base. Most published studies on ERAT are single-center, small-sample retrospective analyses, lacking large-sample, multicenter prospective randomized controlled trials (RCTs). The included cases are mainly limited to simple clinical scenarios, such as acute simple appendicitis and fecalith-related chronic appendicitis. In contrast, there is a paucity of efficacy data regarding complex cases, including pediatric patients, geriatric populations, pregnant women, and patients with comorbidities. These gaps highlight the need for more rigorous and diverse investigations to validate the procedure’s broader clinical utility.

Emerging technologies, such as luminal-adherent self-expanding metal stents for appendiceal drainage, have been reported, and their potential to offer superior outcomes compared with conventional plastic stents represents a key avenue for future research. Optimizing stent design and retention strategies may further enhance the efficacy and safety of ERAT while minimizing complications.
